# COVID-19 and the Heart

**DOI:** 10.14797/mdcvj.1065

**Published:** 2021-12-15

**Authors:** Mouaz H. Al-Mallah

**Affiliations:** 1 Beverly B. and Daniel C. Arnold Distinguished Chair, Professor of Medicine and Cardiology, Weill Cornell Medical College, Director of Cardiovascular PET, Houston Methodist DeBakey Heart & Vascular Center, Houston, TX, USA

**Keywords:** COVID-19, cardiac critical care, ICU, acute circulatory collapse, endotheliopathy, heart failure

For the past 2 years, the world has been living in an ongoing healthcare emergency, namely the coronavirus 2019 (COVID-19) pandemic. Since the earliest days of this unprecedented emergency, cardiovascular care faced numerous challenges that brought significant changes to the management of patients with cardiovascular diseases—with or without COVID-19 infections. More than 24 months into the pandemic, healthcare providers have come a long way in learning about this virus and its associated cardiovascular manifestations. This issue of the *Methodist DeBakey Cardiovascular Journal* reviews the scientific output of the past 2 years, exploring COVID-19 through the lens of how it has impacted cardiovascular care.

In our first article, I, along with Drs. Awad Javaid, Maan Malhafji, and colleagues, review the management of patients with acute chest pain presenting during the COVID-19 pandemic. While explaining how multimodality imaging modalities can be performed safely (for both patients and staff), the review summarizes the evidence behind the utilization of quick and abbreviated procedures during the pandemic. Delving deeper, Drs. Ronak Bahuva, Joe Aoun, and Sachin S. Goel, review the management of acute coronary syndrome in the COVID-19 era. They focus on management of patients presenting with ST-segment elevation myocardial infarction as well as non-ST elevation acute coronary syndrome. They also describe clinical pathways that have impacted the management of these patients during the different COVID-19 surges.

A common dilemma when managing patients with COVID-19 infections is assessing myocardial involvement, especially in the ambulatory setting. A wide range of studies have tested different techniques, such as cardiac magnetic resonance imaging (CMR), including late gadolinium enhancement and T1/T2 overlapping. Drs. Valentino Crudo and Maan Malahfji review the literature describing CMR evaluation of patients with prior COVID-19 infections. This highly controversial topic evokes numerous opinions, depending on the technique and the population studied.

At the heart of it all, intensive care units (ICUs) around the world have faced a seemingly unending flow of patients with COVID-19 and its complications. During local surges, ICUs were frequently full (and sometimes overflowing) while simultaneously battling challenges of limited supplies of personal protective equipment, ventilators, and other ICU essentials. Compounding the problem, ICUs experienced significant staffing shortages among physicians, nurses, respiratory therapists, and other healthcare providers. In their review of the changing face of the ICU during COVID-19, Drs. Atiya Dhala and Faisal Masud explore how ICU staff quickly adapted to deliver safe and appropriate care to the very sick but highly contagious COVID-19 patients. During the surges, many units were staffed by non–intensive-care providers and saw a rapid increase in virtual critical care programs. Additionally, tertiary care centers played a vital role in collaborating with community hospitals to identify critically ill COVID-19 patients who required transfer for advanced therapies.

The COVID-19 pandemic also changed the face of the cardiovascular ICU. Many patients admitted to the ICU had cardiac involvement, often manifesting as troponin elevation as well as left ventricular function depression and hypoxemia. In their review of the most recent evidence on this topic, Drs. Rishi Thaker, Aayush Shah, Ju Kim, and Mahwash Kassi elucidate the role of circulatory support and advanced therapies in patients with COVID-19–infection-associated shock. Mechanical circulatory support, including intra-aortic balloon pump, Impella, and extracorporeal management membrane oxygenation, has been used widely in ICUs for critically ill COVID-19 patients.

One complication that has attracted significant attention is the disturbance in endothelial hemostasis contributing to arterial and venous thrombosis in those with COVID-19. To that end, Drs. John Cooke, John Connor, and Abhishek Jain review the molecular mechanisms that may explain the increase in thromboembolism in these patients, including alteration of endothelial adhesion molecules. Many COVID-19 patients receive anticoagulation and antiplatelets, at least in the acute care setting, to counteract these complications.

Another unique challenge of the pandemic has been managing heart failure, durable left ventricular assist device, and heart transplant patients. Much of this population has delayed care, which is associated with increased cardiovascular mortality and morbidity. Moreover, many elective procedures have been canceled and routine follow-up appointments delayed. In our next article, Dr. Nadia Fida reviews the management of this vulnerable population, addressing challenges of limited resources and minimal access to the patient.

Arrhythmias, both new and existing, have been another challenge for COVID-19 patients. These arrhythmias are usually mediated by the systemic inflammatory response and other acute events, including ischemia, thrombosis and embolism, and hypoxemia. Drs. Summit Pandat, Zhihao Zhu, Stephanie Fuentes-Rojas, and Paul Schurmann, review the spectrum of arrhythmias seen in patients with COVID-19 infections, including postural orthostatic tachycardia and inappropriate sinus tachycardia. They also examine the arrhythmic side effects of some of the COVID-19 infection therapies and describe the long-term effects of COVID-19 on arrhythmias.

Finally, we close this issue with a look forward: What will cardiovascular medicine look like in the aftermath of the worst waves of COVID-19? The past 2 years have undeniably impacted healthcare delivery for cardiovascular patients. Many newer technologies are now widely accepted, available, and used to manage cardiac patients. Although widespread vaccination now offers a glimpse of light at the end of the tunnel, the long-term collateral damage from the pandemic, including prolonged COVID-19 syndrome, continually needs to be addressed. In the closing article of this issue, I examine possible ways forward after so much tragedy.

We hope you find this issue of the journal interesting and informative. While documenting the challenges faced and overcome by the cardiovascular community, we also offer a summary of best practices that can be applied in similar healthcare emergencies. As always, our aim as physicians is to provide efficient and safe care for our patients. We are grateful to our readers for your continued interest and to our expert authors for providing an up-to-date and insightful educational review.

## Editor Biography

The editors of the *Methodist DeBakey Cardiovascular Journal* express our thanks to Dr. Mouaz Al-Mallah for his insight, enthusiasm, and dedication in curating this issue on COVID-19 and the heart.

## Mouaz H. Al-Mallah, MD, MSc

**Figure:**
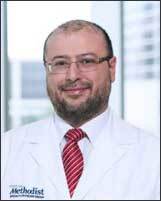


Mouaz H. Al-Mallah, MD, received his medical degree from the American University of Beirut, Lebanon. He completed his training at Henry Ford Hospital, Wayne State University, Brigham and Women’s Hospital-Harvard Medical School, and the University of Michigan, Ann Arbor. He is the director of cardiovascular PET at the Houston Methodist DeBakey Heart & Vascular Center and the associate director of Nuclear Cardiology. He is also the secretary of the American Society of Nuclear Cardiology (ASNC) and will assume the position of vice president on January 1, 2022.

Dr. Al-Mallah started the cardiac PET service at Houston Methodist DeBakey Heart & Vascular Center. This service utilizes the most recent digital PET and performs multiple PET procedures, including PET for myocardial perfusion imaging, viability, assessment of inflammatory cardiomyopathy, and detection of cardiac infections and cardiac devices-related infections as well as vascular graft infections.

He is a member of several American and European cardiovascular councils while serving as an Associate Editor of *Atherosclerosis* and *Cochrane Reviews*. He is also a member of the Editorial Boards of *Journal of the American College of Cardiology* and *European Heart Journal – Cardiovascular Imaging*.

Dr. Al-Mallah has received many local and international awards, including the Francis N. Fernandez Fellowship Award, King Abdullah International Medical Research Center Award in 2011, Best Teacher Award from Henry Ford Hospital, Elite Reviewer for the year 2013 for *the Journal of the American College of Cardiology Journals*, and the King Abdullah International Medical Research Center senior researcher award in 2016. He also received the Houston Methodist DeBakey Heart & Vascular Center Best Teacher Award in 2019 and the center’s Craig Pratt Excellence in Teaching Award in 2020 and 2021. Many of his trainees have established academic careers and received multiple research awards while working under his supervision.

